# An exploration of student experiences of using biology podcasts in nursing training

**DOI:** 10.1186/1472-6920-13-12

**Published:** 2013-01-29

**Authors:** Alison Mostyn, Claire M Jenkinson, Damion McCormick, Oonagh Meade, Joanne S Lymn

**Affiliations:** 1 School of Veterinary Medicine and Science, University of Nottingham, Sutton Bonington Campus, Loughborough, LE12 5RD, UK; 2 Clinical Trial Manager, Nottingham Clinical Trials Unit, University of Nottingham, Nottingham Health Science Partners, C Floor, South Block, Queens Medical Centre, Nottingham, NG7 2UH, UK

**Keywords:** Podcast, Biology, Pre-registration nursing

## Abstract

**Background:**

Students regard biological science as one of the most difficult components of the nursing curriculum. However, a good understanding of this area is essential for effective nursing practice. The aim of this study was to explore nursing students’ perceptions of the usefulness of supplementary biology podcasts for their learning.

**Methods:**

Biological science podcasts (n = 9) were made available to first-year nursing students (n = 189) as supplementary learning tools. On completion of their first year, students were asked to complete a survey which investigated the frequency of their podcast use, reasons for use and their perception of the usefulness of podcasts as a learning tool. 153 of these students participated in the survey study (80.9%). Two focus groups were conducted with students (n = 6) to gain a detailed understanding of student experiences of the usefulness of the podcasts for their learning.

**Results:**

Survey data demonstrated that most students (71%) accessed at least one podcast. The majority of students who reported accessing podcasts agreed that they were useful as learning tools (83%), revision aids (83%) and that they helped promote understanding of course materials (72%). Focus group participants discussed how they found podcasts especially useful in terms of revision. Students valued being able to repeatedly access the lecture materials, and appreciated having access to podcasts from a range of lecturers. Focus group members discussed the benefits of live recordings, in terms of valuing the information gleaned from questions asked during the lecture sessions, although there were concerns about the level of background noise in live recordings. Lack of awareness of the availability of podcasts was an issue raised by participants in both the survey component and the focus groups and this negatively impacted on podcast use.

**Conclusions:**

Nursing students found the availability of biology podcasts helpful for their learning. Successful implementation of these tools to support learning requires teaching staff to understand and promote the importance of these tools.

## Background

The provision of biological sciences education within the pre-registration nursing curriculum has been a source of ongoing debate for more than twenty years [1-5]. Indeed, the move towards emphasising the behavioural sciences within the pre-registration curriculum led to a charge of ‘incomplete holism’ being levelled at nursing education [3]. The suggested reasoning behind the move away from biological sciences is that, in trying to establish itself as an autonomous academic profession, nursing stepped away from the medical model of care with which the biosciences are inextricably linked [1,3]. This has resulted, over time, in a relative disinterest in the biological sciences among nurse educators [2,6] and a lack of bioscience knowledge in students of nursing [7,8]. This problem has resulted in, and been compounded by, both the anxiety this curriculum area provokes within nursing students [7] and the dichotomy between students and lecturers in terms of their views of the relevance of biological sciences to clinical practice [4,7].

A number of recent studies have identified that qualified nurses consider their biological science knowledge to be both weak and insufficient to equip them for their roles on registration [5,9]. The expansion of advanced nursing roles such as non-medical prescribing over recent years demands that even more importance be placed on the development of this understanding during nurse training [10].

While feedback from students has suggested that more time should be allocated to biosciences within the nursing curriculum [4,7], this is unlikely to be achievable in what is an already crowded curriculum. The burden of the nursing curriculum, in common with the medical curriculum, does not easily allow for more hours to be dedicated to a single subject and can provide information overload [11,12]. Perhaps a more pragmatic approach would be to think about more effective utilisation of the time allocated to biology in the curriculum. Indeed, on close reading of the study by Davies and colleagues, the student comments appear to suggest that lecture material needs to be covered more slowly rather than suggesting the inclusion of additional material [4].

Using lecture formats for presentation of biological sciences seems to be preferential for this particular group of students for a number of reasons. Nursing students have been suggested to be ‘pedagogic’ rather than ‘andragogic’ learners who prefer to ‘listen and learn’ rather than actively participate [13]. Similarly, more than 70% of first year nursing students in one study agreed that lectures were an effective means of receiving new information in relation to life sciences [14]. Taking into consideration these preferred learning styles, it might be expected that the use of audio recordings (hereafter referred to as ‘podcasts’) of biological science lectures may be of value to the nursing student population. While a number of studies have reported the value of podcasts in supporting medical education [15-18], less research exists in relation to the use of this technology in nursing education [19-21]. The existing research is also complicated by the trend towards using podcasts as an alternative to face-to-face lectures [19,20], rather than as an additional supporting learning tool [21]. As a supplementary learning tool, podcasts have been found to enhance non-medical prescribing students’ understanding of pharmacology and were associated with improved exam performance [21]. Moreover, students reported that access to these lecture podcasts allowed them to better manage their learning and understanding of complex information [22].

In our previous study, the main author was involved in developing and promoting podcast use to support their personal teaching, and was therefore fully engaged with the project. This current study, however, involved making podcasts available to a wider, and geographically dispersed, student group who were taught by several different lecturers, the majority of whom were not involved in the production of podcasts themselves. The purpose of this study was to explore student experiences of the usefulness of podcasts of biological science lectures to aid learning in first-year nursing students.

## Methods

### Design

Nine live biological science lectures were edited and uploaded to the virtual learning environment WebCT [21]. The podcasts were made available to all first year students on the Diploma/BSc (Hons) nursing programme at the University of Nottingham delivered at the Boston and Derby education centres between September 2009 and January 2010. The lectures were recorded by staff on each of the university teaching sites and podcasts were made available to students across both sites via WebCT.

In order to examine student experiences and perceptions of biology podcasts, a mixed methods approach was used. Firstly, a survey was administered to all first year students at the end of the academic year, which contained both fixed response questions on podcast use and perceptions of the usefulness of podcasts, and open-response questions for students to include further comments. In order to build on the quantitative and qualitative data gained from the survey data, a further qualitative component was added to the student evaluation process by inviting students to attend focus groups to discuss their experience of the usefulness of the podcasts for their learning.

### Participants

The survey was distributed to all first year diploma/BSc nursing programme students who were enrolled on the ‘Biological Sciences Applied to Nursing’ module between September 2009 and January 2010. The survey was distributed at the end of the academic year. All students were also contacted, by email, and asked if they would consent to take part in focus groups. All students who expressed an interest were invited to attend a focus group. The sampling strategy for the study was therefore a convenience sample. While all students who had access to the podcasts were invited to participate in the survey and focus groups, the final sample reflected those who were willing and available to take part.

### Survey method

A survey, based on the tool previously used by the authors to evaluate the usefulness of supplementary podcasts in non-medical prescribing [21], was used to examine student podcast use and their perceptions of the usefulness of podcasts for their learning. All students who had attended the ‘Biological Sciences Applied to Nursing’ module were invited to complete the questionnaire, which was distributed to students at the end of the module. Completed questionnaires were voluntarily returned directly to the lecturer following the lecture or indirectly via internal mail.

### Focus group method

Two focus groups were carried out. A focus group topic guide was developed to explore, in more detail, student experiences of the barriers and facilitators to podcast use, reasons for use and perceived advantages and disadvantages of podcasts for developing learning. This guide was used flexibly in order to facilitate a participatory approach.

All students from the Derby and Boston centres who had access to the podcasts were invited to participate in the focus groups. Six students volunteered to take participate. All volunteers were students attending the Boston education centre as part of the September 2009 (n = 3) and January 2010 cohorts (n = 3). Each focus group was made up of one male and two female students. Three students were aged 41–50, two were aged 31–40 and one was aged 25–30. All students who attended the focus groups had used the podcasts.

Both focus groups were conducted in a private room at the University over a lunchtime period with refreshments being provided. The focus groups were conducted by a researcher who was independent from the students’ teaching team and a second independent researcher from the School of Nursing acted as an observer and took notes with respect to body language and group interactions. The focus groups lasted for 90 minutes each. Discussions were digitally recorded using an MP3 recording kit and the recordings were transcribed verbatim.

### Data analysis

Questionnaire data were analysed using SPSS for Windows v16 (Chicago, IL, USA). For descriptive purposes, frequencies, means and standard deviations were presented. For dichotomous variables, the chi-square test for association was performed to compare response by age group and sex. The five-point Likert scale in the questionnaire was analysed as non-parametric data using the Mann-Whitney U-test to compare responses by age group and by sex.

Focus group transcripts were analysed independently by two members of the research team using a framework analysis technique [23,24]. Briefly, both researchers initially read through the transcripts, then read through again, highlighting, cutting and pasting sections which contained one or more discrete themes. Further re-reading and grouping of the identified themes into “key” themes or categories reduced the number of themes and highlighted overarching “super-themes” under which sub-themes were clustered [24]. The two researchers met to discuss the key themes which emerged from reading of the transcripts.

### Ethical approval

This study was classified as a teaching evaluation by the research ethics officer within the School of Nursing, Midwifery & Physiotherapy, and as such, did not require specific ethics approval.

## Results

### Survey data

#### Response rate

The response rates across study sites are presented in Table 1. Surveys were distributed to 189 students. The total response rate to the questionnaire was 81% (n =153) and the individual response rates from the Derby and Boston centres were 73% and 91% respectively.

**Table 1:** Survey response rate

**Study site**	**Invited to complete survey**	**Completed survey**	**Response rate**
Boston	104	76	91%
Derby	85	77	73%
Both sites	189	153	81%

Table 1 presents the total survey response rate and the survey response rates across study sites.

#### Demographic characteristics

Demographic characteristics are summarised in Table 2. The majority of participants were aged 17 to 24 years (60%). The number of respondents from each study site was almost equal (n = 77 Derby, n =76 Boston). All respondents had access to a home computer with internet access and most (76%) owned or had access to an MP3 player or iPod. Students aged less than 25 years were significantly more likely to own or have access to an MP3 player or iPod than older students (82% v 67% respectively, χ^2^ (1) = 4.096; p = 0.043). Forty-three percent of respondents (n = 66) rated their level of confidence with internet-based technologies as being high or very high and the youngest (17–24 years) respondents were more likely than older respondents (≥ 25 years) to do so (54% v 28% respectively, χ^2^ (1) = 10.031; p = 0.002).

**Table 2:** Demographic Characteristics of Survey Respondents

	***n***	***Percentage***
**Age**		
*17-24*	92	60%
*25-40*	49	32%
*41+*	12	8%
**Study site**		
*Derby*	77	50%
*Boston*	76	50%
**Home computer & internet access**		
*Yes*	153	100%
**MP3 player ownership**		
*Yes*	116	76%
*No*	37	24%
**Comfort level with internet-based technologies**		
*High or Very High*	66	43%
*Very low - Average*	87	57%

Table 2 presents demographic details for survey respondents including age, study site, internet access, MP3 ownership and comfort level with internet-based technologies.

#### Podcast use and accessibility

One hundred and nine students (71%) accessed the podcasts. Most students (59%) reported using them one to three times with 17% using them eight or more times. Despite the fact that 79 (73%) of those who used the podcasts had access to an MP3 player, most indicated that they listened to podcasts directly through their computer (70%). Only 6% downloaded the podcasts and listened to them on an MP3 player or iPod, and a further 16% used a combination of both methods. Nine students (6%) reported difficulty accessing the podcasts, all of whom were students at the Derby centre. The reasons for this were reported as a lack of awareness of podcast availability and difficulty locating podcasts on WebCT.

#### Reasons for podcast use

**Figure 1:**
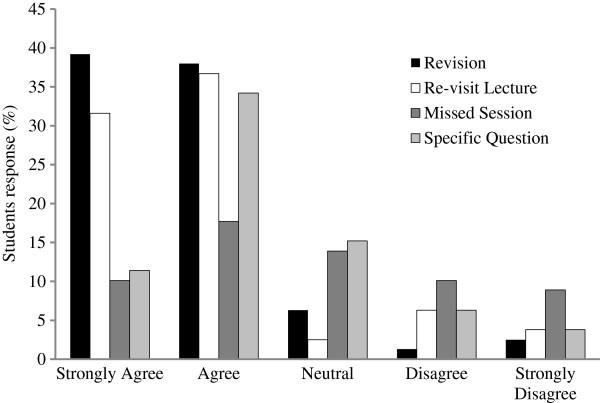
**Reasons for podcast use.** Nursing students (n = 109) were asked to rate their use of the biological science lecture podcasts for revision purposes, to re-visit the lecture generally, because they missed the session and ‘in order to answer a specific question’ on a scale of 1 to 5 with 1 representing ‘strongly agree’ and 5 representing ‘strongly disagree’.

Of the 109 (71%) students who used podcasts, the majority agreed or strongly agreed that they used them to revisit a lecture (73%) and when they needed to revise (83%). Fewer students used them when they had a specific question (45%) or when they missed a session (30%; Figure 1). Of the students who used them when they had a specific question, most (81%) stated that they always or usually found the answer they were looking for.

#### Perception of Podcasts

**Figure 2:**
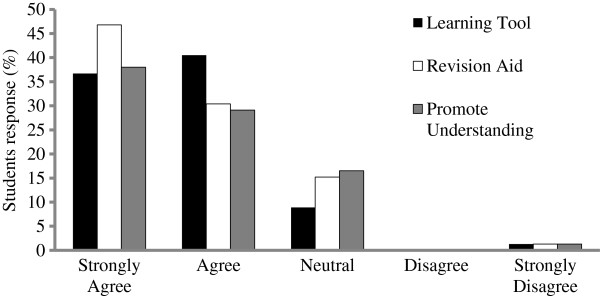
**Student perceptions of the usefulness of podcasts.** Nursing students (n = 109) were asked to rate their perception of the usefulness of the biological science lecture podcasts as a learning tool, a revision aid and to promote understanding of the subject on a scale of 1 to 5 with 1 representing ‘strongly agree’ and 5 representing ‘strongly disagree’.

In terms of students’ perceptions of the usefulness of the biological sciences lecture podcasts, the majority of respondents agreed or strongly agreed that podcasts were a useful learning tool (83%), revision aid (83%) and helped promote understanding of course materials (72%; Figure 2).

Thirty-two respondents (21%) provided open text comments regarding further feedback about the use of podcasts, 53% of which provided positive evaluations of their experiences of podcasts.

“Not enough of them. Ideal as a revisit to lectures around exam time.”

“Easy to use, I only had average skills and could do it. Loved being able to listen again and again until I understood.”

The main negative comment made by students in this open text box was concerned with a lack of knowledge of the availability of the podcasts:

“I would have used them earlier had I realised that they were there, or maybe listened if somebody had told me.”

Other negative comments related to technical difficulties some students experienced in downloading the podcasts (e.g., *“some wouldn’t download to MP3 player”*) and noise issues relating to the use of live lecture recordings (e.g., “*just the sound quality could be improved i.e. coughing/talking etc. during the podcast”*).

### Focus groups

#### Focus group themes

The focus group provided richer detail on the students’ use of, and perception of the usefulness of the podcasts of biological sciences lectures. The two overarching themes which emerged from analysis of both focus groups were that of ‘facilitating learning’ and ‘barriers to podcast use’. While there was general agreement between the two focus groups in terms of the emerging sub-themes, there were two sub-themes which emerged from only one of the focus groups (Table 3).

**Table 3:** Themes emergent from analysis of focus group data

**Overarching Theme**	**Sub -theme**	**FG1**	**FG2**
**Facilitating Learning**	Revision tool	X	X
	Repetition	X	
	Aural learning		X
	Benefit of other lecturers	X	X
	Benefit of live lecture recording	X	X
	Extra learning opportunities	X	X
**Barriers to podcast use**	Access difficulties	X	X
	Late identification	X	X

Table 3 presents overarching themes and sub-themes that emerged from the analysis of focus group data.

#### Facilitating learning

The main use of the podcasts which emerged from all students across both focus groups was as a revision tool:

‘...*it was a case of looking at it from several different ways instead of always just opening your textbooks and looking at your revision notes, finding a third way of doing the revision and I found that really useful’* (Focus group 2).

In relation to how the podcasts helped facilitate learning, the theme which emerged from all students in Focus Group 1 was that of the benefit of repetition:

*‘I find personally I learn through repetition which is why I do like podcasts because you can replay it and replay it until you are happy, you know it is not like a lecture that’s been done and gone.’* (Focus group 1).

An additional element of facilitating learning which students discussed in Focus Group 2 only, was how some students had a preference for aural learning. They then felt that this type of learning tool better facilitated their learning style:

*‘I think I learn better from listening so they’re quite useful.’* (Focus group 2).

While it may have been expected that students would have had a preference for listening to podcasts recorded by their own lecturer, students from each focus group identified the availability of podcasts from different lecturers as being an added benefit of the podcasts:

*‘When you hear someone else talk about something as well from a slightly different perspective you can thing ‘oh yeah actually I can understand that’ and so that was quite useful as well actually.’* (Focus group 2).

In contrast to some of the comments from the open text boxes in the survey, students who participated in the focus groups felt that the fact that the podcasts were audio recordings of live lectures had additional benefits without impacting negatively on the classroom atmosphere. For example, listening to the questions posed by students during the live lecture recordings was seen as beneficial by one participant:

*‘Yeah I don’t think it’s a bad thing that people ask questions because chances are I might have wanted to ask that question myself and someone’s done it for me.’* (Focus group 2).

Despite the majority of students indicating in the survey data that they listened to the podcasts through a computer, one student from each of the focus groups identified using the podcasts in a mobile format which enhanced their available learning opportunities:

*‘So you just go on your phone and download it and wherever you go you take your phone so you can listen to it in the car or if you are doing something at home you can put it on and listen to it again and you are just absorbing that information.’* (Focus group 1).

#### Barriers to podcast use

As identified in the survey open-ended responses, a barrier to student use of the podcasts was related to not being aware that the podcasts were available. The delay in the introduction of the podcasts to students by lecturing staff was discussed as a barrier to use by participants in both focus groups. The participant below describes how she would have preferred to have been informed about the podcasts earlier in the academic year:

*‘I think it would have been nice to have had it in my knowledge so I could have started back in the beginning of the year to have kind of gone over. Maybe they were there and I didn’t know about them though.’* (Focus group 2)

A further barrier to accessing the podcasts identified by those in the focus group was technical issues with accessing the podcasts, particularly in relation to locating and downloading the podcasts:

*‘I think for me personally it wasn’t something that I used initially because at that time I was still familiarising myself with navigating around WebCT … it can be quite difficult to navigate round.* (Focus group 1).

## Discussion

The purpose of this study was to explore nursing students’ experience of using podcasts as a support to learning in relation to biological sciences. The data indicate that the majority of students accessed the podcasts and perceived them to be useful as a learning tool, which compares well with previous literature data in terms of both medical and nursing education [15-18,20,21]. The ability to build biological science knowledge by repetition was identified in both the survey and the focus group components. Repetition has previously been reported as one of the main benefits of podcasts in developing pharmacology knowledge in non-medical prescribing students [22]. Students in this study identified the use of podcasts as a helpful revision tool, which is consistent with literature data from other student groups [16,21,22].

In contrast to our previous study, where 91% of non-medical prescribing students used pharmacology podcasts [21], only 71% of students in this group used the biology podcasts. Whilst it may be tempting to think that this reduced uptake is due to the different learning needs of the student groups studied, it is likely that this difference may also relate to feedback from some participants in the survey and focus groups that they were not made aware of the availability of the podcasts at the beginning of the course. This late introduction of podcasts to students by lecturers highlights the importance of having complete ‘buy-in’ of faculty staff in relation to the use of technology such as podcasts to enhance student learning. A number of the lecturers involved in teaching the students in this study did not record their own lectures and as such, may not have been fully ‘signed up’ to making students aware of the availability of these lecture podcasts. This is in contrast to our previous study where the main author was involved in producing pharmacology podcasts and introducing these podcasts to students in her pharmacology course [21,22].

The late introduction of podcasts to students in this study may also have resulted from a lack of awareness of the potential impact of this type of technology on the development of student knowledge and understanding. This may itself be the result of a lack of confidence among lecturers about using podcast technology. Indeed, a survey of nurse educators in Sweden indicated that less than 50% believed they had sufficient IT skills to fulfil their role [25]. Similarly, a study of nurse educators in the USA identified that they suffer from ‘technological stress’ which was associated with a lack of knowledge around how to use technology in the classroom [26]. These issues are exacerbated by the differences in learning experiences of faculty members who, for the most part, were born before the existence of digital technology and can at best be described as ‘digital immigrants’ compared with the new generation of students who have grown up with technology, the ‘digital natives’ [27]. It must be emphasised, however, that the pedagogical impact of technology is only slowly emerging from the literature and hence development of an understanding of its importance by faculty staff will necessarily follow at a delayed rate.

In order to promote staff engagement with these learning tools, training could be provided to staff. This training could involve presenting staff with the emerging evidence related to the benefits of these tools for student learning as well incorporating guidance to help staff use these tools in their own teaching. The feedback from students in this study suggests that listening to live recordings was beneficial in terms of hearing questions asked by students and listening to podcasts from multiple lecturers. This feedback is useful for educators as it may mean that there is less need to commit extra time to producing podcasts in a studio environment. Improvements, however, may still need to be made in recording lectures to minimise the disruption from any background noise.

While technological difficulties in accessing podcasts were a barrier to podcast use, these difficulties are not unique to this group of students, as identified in our previous studies [21,22]. Indeed, since the publication of our previous work, the non-medical prescribing teaching team have worked hard to resolve the majority of these technical issues. In terms of moving forward with incorporating podcasts into the learning resources for students, it is important that students receive adequate training and support to equip them to use these learning tools. Training could involve a short presentation prior to module commencement, demonstrating to students how to locate and download the podcasts from the VLE. Similarly, a list of FAQs about locating and downloading the podcasts could be placed within the VLE for students’ reference.

A limitation of this study is that all of the focus group participants were podcast users and therefore the researchers did not have the opportunity to further investigate students’ reasons for not using podcasts. The low number of students taking part in the focus group may have been due to poor timing. The focus groups took place at a time when students had completed the majority of their lectures and were on placement. It may therefore have been more useful to conduct the focus groups while students were still attending lectures to encourage more participation.

One of the ongoing questions surrounding the use of podcasts is whether these represent a supplement, or an alternative, to lectures. Students in this study expressed the opinion that live lecture recordings had additional benefits, suggesting that they saw podcasts as a supplement to lectures and not as an alternative. This desire for the face-to-face lecture component is consistent with data from other studies [19,21,28] and suggests that whilst podcasts can help develop student knowledge and understanding of biological sciences, they should be only be considered as an addition to traditional lecture provision.

The results of this study are consistent with previous literature in terms of student perceptions of the benefits of podcasting to their learning. This study does however highlight the potential barriers to incorporating podcasting technology more widely across the nursing curriculum. Whilst students have bought-in to these technologies, they cannot be fully effective without the complete engagement of teaching staff.

## Conclusions

The most important message that can be taken from this study is that nursing students find the availability of podcasts helpful in constructing their biological sciences knowledge. ‘Buy-in’ from educators is required, however, to ensure that students can make the most of these learning tools to support the development of their learning. In terms of facilitating staff ‘buy-in’, institutional training could be provided to highlight the emerging evidence for the impact of information technology on current pedagogy and to help educators understand how to incorporate these technological tools into their teaching. Providing training to students in terms of locating and downloading podcasts may also prevent some of the technological barriers reported in this study from affecting students’ engagement with these learning tools.

## Competing interests

The authors declare that they have no competing interests.

## Authors’ contributions

JSL and AM conceived of, designed the study and obtained the funding. DM produced the biological science podcasts. OM facilitated the focus groups. AM, CJ and JSL analysed the data. CJ, OM and JSL drafted the manuscript. All authors have read and approved the final manuscript.

## Pre-publication history

The pre-publication history for this paper can be accessed here:

http://www.biomedcentral.com/1472-6920/13/12/prepub
